# Two-year neurodevelopmental outcome in preterm neonates with cerebral oxygenation monitoring after birth: a multinational, multicenter retrospective follow-up study of the COSGOD III trial

**DOI:** 10.3389/fped.2026.1754084

**Published:** 2026-06-15

**Authors:** Gerhard Pichler, Christina H. Wolfsberger, Katharina Goeral, Marlene Hammerl, Tina Perme, Eugene M. Dempsey, Laila Springer, Gianluca Lista, Tomasz Szczapa, Hans Fuchs, Jenny Bua, Alexander Avian, Renate Fuiko, Ursula Kiechl-Kohlendorfer, Lilijana Kornhauser Cerar, Iyshwarya Stapleton, Kriszta Molnar, Ilaria Stucchi, Roksana Malak, Bernhard Schwaberger, Katrin Klebermass-Schrehof, Nariae Baik-Schneditz

**Affiliations:** 1 Division of Neonatology, Department of Pediatrics and Adolescent Medicine, Medical University of Graz, Graz, Austria; 2 Research Unit for Neonatal Micro- and Macrocirculation, Medical University of Graz, Graz, Austria; 3 Comprehensive Center for Pediatrics, Department of Pediatrics and Adolescent Medicine, Division of Neonatology, Intensive Care and Neuropediatrics, Medical University of Vienna, Vienna, Austria; 4 Department of Pediatrics II, Neonatology, Medical University of Innsbruck, Innsbruck, Austria; 5 NICU, Department for Perinatology, Division of Gynaecology and Obstetrics, University Medical Centre Ljubljana, Ljubljana, Slovenia; 6 INFANT Research Centre, University College Cork, Cork University Maternity Hospital, Cork, Ireland; 7 Department of Neonatology, University Children’s Hospital of Tübingen, Tübingen, Germany; 8 Neonatologia e Terapia Intensiva Neonatale (TIN) Ospedale dei Bambini “V.Buzzi”, Milano, Italy; 9 II Department of Neonatology, Neonatal Biophysical Monitoring and Cardiopulmonary Therapies Research Unit, Poznan University of Medical Sciences, Poznan, Poland; 10 Divisual of Neonatology and Pediatric Intensive Care Medicine, Center for Pediatrics and Adolescent Medicine, Medical Center - University of Freiburg, Faculty of Medicine, University of Freiburg, Freiburg, Germany.; 11 Neonatal Intensive Care Unit, Institute for Maternal and Child Health, IRCCS “Burlo Garofolo”, Trieste, Italy; 12 Institute for Medical Informatics, Statistics and Documentation, Medical University of Graz, Graz, Austria

**Keywords:** cerebral tissue oxygen saturation, near-infrared spectroscopy, neonatal resuscitation, neurodevelopmental outcome, preterm neonates

## Abstract

**Introduction:**

During immediate neonatal transition, cerebral tissue oxygen saturation (crSO_2_) exhibits different behavior compared to peripheral arterial oxygen saturation (SpO_2_). Lower crSO_2_ levels have been linked to a higher risk of intraventricular hemorrhages and adverse outcomes in preterm neonates.

**Aim:**

To assess mortality and long-term neurodevelopmental outcomes at 2 years of age in preterm neonates who underwent cerebral oxygen saturation monitoring and received targeted interventions during the immediate postnatal transition.

**Methods:**

This 2-year follow-up observational study included preterm neonates born before 32 weeks of gestation and originally enrolled in the multicenter, multinational COSGOD III randomized controlled trial across 10 European centers. Neonates who underwent routine neurodevelopmental assessments at 18–30 months of corrected age or had available outcome data from medical records were included. Analyses were conducted according to the COSGOD III group allocations [near-infrared spectroscopy (NIRS) group vs. control group]. Subgroup analyses were performed for very preterm (28 + 0 to 31 + 6 weeks) and extremely preterm (<28 + 0 weeks) neonates. The primary outcome was survival without moderate-to-severe neurodevelopmental disability (NDD), defined by cognitive or language impairment, cerebral palsy, visual, or hearing impairment.

**Results:**

Of the 526 eligible neonates, 417 were analyzed. Survival without moderate-to-severe NDD was similar between the groups: 129 (61.1%) in the NIRS group and 128 (62.1%) in the control group [(relative risk) (95% CI): 0.98 (0.86–1.21), *p* = 0.761]. No significant difference in survival without moderate-to-severe NDD was observed between the NIRS group and control group in the extremely (<28 weeks) low gestational age subgroups. However, among very (28 + 0 to 31 + 6 weeks) preterm neonates, the NIRS group showed a lower incidence of cerebral palsy, visual impairment, and/or hearing impairment (0.9%) than the control group (6.9%) [RR (95% CI): 0.12 (0.29–0.45), *p* = 0.002].

**Conclusions:**

Cerebral oxygen saturation monitoring with targeted interventions during immediate postnatal care did not improve overall survival without NDD but may reduce specific impairments in very preterm infants (28 + 0 to 31 + 6 weeks).

## Introduction

The immediate neonatal transition is a complex physiological process. Current and recent neonatal resuscitation guidelines recommend the monitoring of peripheral arterial oxygen saturation (SpO_2_) and heart rate to guide respiratory support and oxygen titration immediately following birth (1, 2). However, during the immediate neonatal transition, SpO_2_ exhibits different behavior compared to cerebral tissue oxygen saturation (crSO_2_) monitored using near-infrared spectroscopy (NIRS) (3, 4). Studies conducted in delivery rooms have observed that neonates who developed intraventricular hemorrhages (IVH) exhibited lower crSO_2_ values, falling below the reference ranges, compared to those who did not develop IVH (5–7). Furthermore, preterm neonates with adverse outcomes had, in addition to lower gestational age, lower crSO_2_ values during the immediate transition compared to preterm neonates with age-appropriate outcomes (8). A prospective randomized controlled pilot feasibility study (COSGOD phase I/II trial) at two tertiary-level neonatal intensive care units (Graz/Austria, Edmonton/Canada) (9) reported a 55.4% relative reduction in cerebral hypoxia within the first 15 min after birth, when crSO_2_ monitoring was used in addition to SpO_2_ to titrate supplemental oxygen and guide respiratory support. Based on these findings, a multinational, multicenter, randomized controlled phase III trial (COSGOD III) was performed in 11 centers across Europe and Canada (10, 11). Preterm neonates born before 32 weeks of gestation were randomly assigned to either standard care (control group) or standard care plus crSO_2_ monitoring with dedicated interventions (NIRS group) during immediate transition (first 15 min after birth) and resuscitation. The primary outcome was defined as a composite of survival without cerebral injury, characterized by the absence of any IVH and/or cystic periventricular leukomalacia at term-equivalent age or before discharge. The COSGOD phase III trial demonstrated no substantial difference in survival without cerebral injury between the NIRS group and control group, with a 4.3% non-significant higher survival without cerebral injury in the NIRS group. No serious adverse reactions or serious adverse device-related events were observed. However, the effects of clinical care with crSO_2_ monitoring-guided interventions during the immediate transition, as described in the COSGOD III trial, on long-term outcomes remain uncertain (10, 11). Therefore, long-term outcome data from neonates included in the COSGOD III trial are of great interest.

This study aimed to analyze survival without moderate-to-severe neurodevelopmental disability (NDD) in neonates enrolled in the COSGOD III trial. We hypothesized that preterm neonates who underwent cerebral oxygenation monitoring with dedicated interventions during resuscitation after birth would show better survival and neurodevelopmental outcomes at a corrected age of 2 years (18–30 months) compared to neonates in the control group with standard care.

## Methods

A retrospective study was conducted as a 2-year follow-up to the COSGOD III trial (10, 11). The detailed methods of the COSGOD III trial are described elsewhere (10, 11). The COSGOD III trial was conducted across 11 centers in Europe and Canada and included preterm neonates born before 32 weeks of gestation. A total of 1,121 neonates were screened for eligibility to participate in the COSGOD III trial prenatally, and 655 neonates were randomized between 31 December 2017, and 30 October 2021. The primary outcome, survival without cerebral injury at term age or before discharge, was analyzed in 607 neonates, of whom 303 were included in the NIRS group and 304 in the control group. The 2-year follow-up study was approved by the local ethics committee at each center. This study was registered at ClinicalTrials.gov (NCT06141733).

### Inclusion and exclusion

Preterm neonates included in the COSGOD III trial were eligible for this 2-year follow-up. Neonates who underwent routine neurodevelopmental outcome assessments at 2 years, or for whom outcome data were available from their medical history at 2 years (18–30 months) of corrected age, were included. Centers with follow-up data available for fewer than 50% of the included neonates in the COSGOD III trial were excluded *a priori* from the present follow-up study, aiming to minimize the impact of missing data while avoiding excessive loss of available information.

### Outcome measures

The primary outcome was a composite of survival without moderate-to-severe NDD at 2 years (18–30 months) of the corrected age. To assess survival without moderate-to-severe NDD, the two primary outcome parameters were death and moderate-to-severe NDD. A child was classified as having moderate-to-severe NDD if any of the following four conditions were present: (i) cognitive or language disability assessed by a standardized developmental assessment [Bayley Scales of Infant Development (BSID) II test with cognitive scale score <85, BSID III/IV test with cognitive or language composite scale score <85, or Parent Report of Children's Abilities-Revised (PARCA-R) non-verbal cognition or language score <70] or, if no standardized developmental assessment was available, informal assessment from the patients’ medical history demonstrating inability to speak more than five words; (ii) cerebral palsy was assessed by Gross Motor Function Classification Score (GMFCS) ≥ 2 or if no GMFCS was available, informal assessment from the patients’ medical history demonstrating that the child was not able to walk alone; (iii) visual impairment defined as an impairment even with glasses or only being able to perceive light or being blind (in one or both eyes); and (iv) hearing impairment defined as hearing loss requiring hearing aids or complete deafness.

For an infant to be included in further analyses, all four conditions had to be met.

### Data assessment

Two independent research fellows collected and clarified discrepancies at each center. Outcome data at 2 years of corrected age were anonymized at each center participating in this ancillary study using the local patient ID used in the COSGOD III trial.

### Blinding

In the COSGOD III trial, group allocation was documented only in the investigators’ log files and the (electronic) case report form. In the patients’ medical charts, only participation in the COSGOD III trial was documented. During the assessment of the 2-year neurodevelopmental outcome, the assessors did not have access to the investigators’ log files and (e)CRF.

### Analyses

The baseline characteristics of neonates are presented as mean and standard deviation or median and interquartile range for continuous data, and numbers with percentages for categorical data. Baseline characteristics were compared using linear mixed models (random effect: centers) for continuous data, and generalized linear models (random effect: centers) for categorical data. To answer the hypothesis of whether survival without moderate-to-severe neurodevelopmental disability (yes vs. no) differs between neonates in the control and intervention groups, generalized linear models (probability distribution: binomial; link function: log; random effect: center) were used, and adjusted RR with 95% CI were estimated.

The comparison of secondary outcome data was exploratory and, therefore, no correction for multiple testing was performed.

Comparable analyses were performed for subgroups of very preterm neonates (28 + 0 to 31 + 6 weeks of gestation) and extremely preterm neonates (<28 weeks of gestation). Owing to significant baseline differences in short-term neonatal outcome culture-proven sepsis until term-corrected age between the NIRS group and control group among preterm neonates <32 weeks, which may influence long-term outcomes, the long-term analyses for this group were adjusted for infectious short-term morbidities (sepsis including necrotizing enterocolitis).

## Results

**Figure 1:**
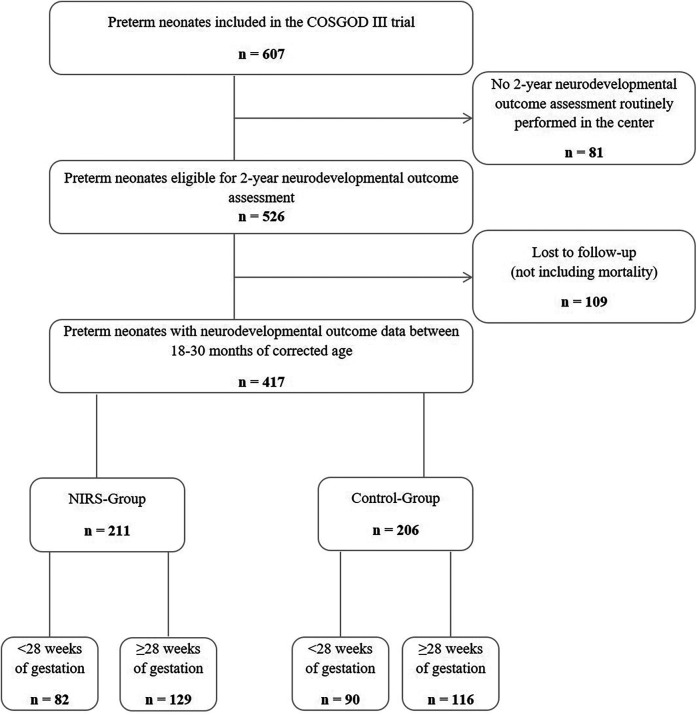
Inclusion–exclusion flow chart.

From the 607 neonates included in the COSGOD III trial, 526 (86.7%) neonates were eligible for the present 2-year follow-up study. One center (Edmonton, Canada) was excluded *a priori* because no follow-up data were available. Among the 526 eligible neonates from the remaining 10 centers, no follow-up data were available for 109 neonates, resulting in a follow-up rate of 79.2% (*n* = 417) with 172 (41%) extremely preterm (<28 weeks) neonates and 245 (59%) very preterm (28 + 0 to 31 + 6 weeks) neonates (Figure 1). Neonates, who were lost to follow-up, had a higher gestational age than those included in the present study (Supplementary Table 1). Assessment of the 2-year follow-up outcome was based on 24 (6%) neonates on mortality, 352 (84%) neonates on 2-year neurodevelopmental follow-up visits in outpatient clinics, and 41 (10%) neonates on informal assessment of the patients’ medical history. For standardized developmental assessment, only the BSID III was used.

Overall, there were no significant differences in the demographic data at 2 years of corrected age between the NIRS group and control group. Neonates in the NIRS group had a median gestational age (IQR) of 28.7 (26.9–30.6) weeks, compared to 28.4 (26.3–30.1) weeks in the control group (*p* = 0.138; Supplementary Table 2).

The primary outcome of the present study, survival without NDD at 2 years of corrected age, showed no significant difference between the NIRS group and control group (Table 1). In the NIRS group, there were significantly fewer neonates with hearing loss, although the absolute numbers were very low, with one in the NIRS group and two in the control group. Morbidities at term age or before discharge in the present cohort were not significantly different between the NIRS group and control group, except for a lower incidence of culture-proven sepsis in the NIRS group. To address this discrepancy, 2-year follow-up data were adjusted for culture-proven sepsis; however, no significant differences were found between the NIRS group and control group.

**Table 1:** Short-term and long-term outcome of preterm neonates <32 weeks of gestation with and without monitoring of cerebral oxygen saturation to guide interventions during immediate transition after birth.

	NIRS-group *n* = 211	Control-group *n* = 206	Relative Risk (95% CI)	*p*-value
Long-term outcome at 2 years corrected age (adjusted for culture proven sepsis)				
Survival without moderate-to-severe NDD (*n* = 257), *n* (%)	129 (61.1)	128 (62.1)	0.95 (0.82—1.10)	0.495
Death or moderate-to-severe disability (*n* = 159), *n* (%)	82 (38.9)	77 (37.4)		
Postnatal accident with cerebral injury (*n* = 1), *n* (%)	–	1 (0.5)		–
Death (*n*=24), *n* (%)	12 (5.7)	12 (5.8)	1.07 (0.61—1.91)	0.805
Moderate-to-severe disability (*n* = 135), *n* (%)	70 (33.2)	65 (31.6)	1.06 (0.79—1.43)	0.691
Cerebral palsy, yes/no, *n* (%)	5/162 (3.1)	7/157 (4.5)	0.74 (0.25—2.24)	0.600
Vision impairment, yes/no, *n* (%)	4/162 (2.5)	9/157 (5.7)	0.45 (0.10—2.24)	0.340
Corrected hearing loss/deaf, yes/no, *n* (%)	2/162 (1.2)	3/157 (1.9)	0.68 (0.48—0.97)	0.032
Cerebral palsy and/or vision impairment and/or corrected hearing loss/deaf, yes/no, *n* (%)	6/162 (3.7)	12/157 (3.8)	0.52 (0.17—1.58)	0.246
Bayleys cognitive score, median (IQR)	100 (90—110, *n* = 173)	100 (85—105, *n* = 173)		0.310
Bayleys language score, median (IQR)	97 (79—109, *n* = 115)	97 (84—109, *n* = 117)		0.703
Age corrected, months, median (IQR)	24 (24—26, *n* = 198)	24 (24—26, *n* = 194)		0.155
Weight, kg, median (IQR)	12 (11—13, *n* = 157)	12 (11—13, *n* = 152)		0.939
Height, cm, median (IQR)	87 (84—90, *n* = 154)	87 (84—90, *n* = 150)		0.798
Head circumference, cm, median (IQR)	48 (47—49, *n* = 150)	48 (47—49, *n* = 151)		0.622
Short-term outcome at term age or before discharge				
Survival without cerebral injury*, *n* (%)	173 (83.4)	161 (78.2)	1.07 (0.98—1.16)	0.135
Death, *n* (%)	12 (5.7)	10 (4.9)	1.17 (0.61—2.23)	0.630
No intraventricular hemorrhage, *n* (%)	184 (87.2)	169 (82.0)		0.132
Intraventricular hemorrhage I–II, *n* (%)	17 (8.1)	26 (12.6)		
Intraventricular hemorrhage III–IV, *n* (%)	10 (4.7)	11 (5.4)		
No cystic periventricular leukomalacia, *n* (%)	207 (98.1)	203 (98.5)		0.595
Cystic periventricular leukomalacia II, *n* (%)	2 (1.0)	2 (1.0)		
Cystic periventricular leukomalacia III, *n* (%)	2 (1.0)	1 (0.5)		
Culture proven early onset sepsis, *n* (%)	64 (30.3)	80 (38.8)	0.78 (0.64—0.96)	0.019
Necrotizing enterocolitis any grade**, *n* (%)	12 (5.7)	8 (3.9)	1.46 (0.62—3.46)	0.384
Bronchopulmonary dysplasia^†^, *n* (%)	37 (17.5)	39 (18.9)	0.93 (0.65—1.32)	0.674
Retinopathy of prematurity ≥ Grade 2, *n* (%)	23 (10.9)	30 (14.6)	0.75 (0.55—1.01)	0.058

* Defined as any intraventricular hemorrhage and/or cystic periventricular leukomalacia at term age or before discharge.

† Defined as oxygen dependency or need of respiratory support at 36 weeks corrected age.

** Medical and/or surgical intervention.

Subgroup analyses of extremely preterm (<28 weeks) neonates showed no differences in baseline demographic data at 2 years of corrected age, with neonates having a median (IQR) gestational age of 26.3 (25.3–27.1) weeks in the NIRS group and 26.1 (25.3–26.9) in the control group (*p* = 0.345; Supplementary Table 3). There were no significant differences in survival without NND or in any other outcome measures between the NIRS group and control group at 2 years of corrected age (Table 2).

**Table 2:** Short-term and long-term outcome of preterm neonates <28 weeks of gestation with and without monitoring of cerebral oxygen saturation to guide interventions during immediate transition after birth.

	NIRS-group *n* = 82	Control-group *n* = 90	Relative Risk (95% CI)	*p*-value
Long-term outcome at 2 years corrected age				
Survival without moderate-to-severe NDD (*n* = 75), *n* (%)	39 (47.6)	46 (51.1)	0.93 (0.73—1.19)	0.571
Death or moderate-to-severe disability (*n* = 87), *n* (%)	43 (52.4)	44 (49.9)		
Death (*n* = 21), *n* (%)	10 (12.2)	11 (12.2)	1.02 (0.58—1.80)	0.940
Moderate-to-severe disability (*n* = 66), *n* (%)	33 (40.2)	33 (36.7)	1.12 (0.82—1.54)	0.478
Cerebral palsy, yes/no, *n* (%)	4/50 (8.0)	1/54 (1.9)	4.32 (0.39—48.41)	0.235
Vision impairment, yes/no, *n* (%)	3/50 (6.0)	3/51 (5.6)	1.08 (0.13—9.17)	0.944
Corrected hearing loss/deaf, yes/no, *n* (%)	1/50 (2.0)	0/54 (0.0)	–	–
Cerebral palsy and/or vision impairment and/or corrected hearing loss/deaf, yes/no, *n* (%)	5/50 (10.0)	4/54 (7.4)	1.35 (0.29—6.27)	0.702
Bayleys cognitive score, median (IQR)	93 (75—110, *n* = 56)	95 (80—105, *n* = 69)		0.944
Bayleys language score, median (IQR)	91 (72—106, *n* = 37)	91 (74—103, *n* = 46)		0.978
Age corrected, months, median (IQR)	26 (24—28, *n* = 72)	24 (24—27, *n* = 79)		0.073
Weight, kg, median (IQR)	11 (10—12, *n* = 50)	11 (11—12, *n* = 54)		0.688
Height, cm, median (IQR)	86 (83—90, *n* = 50)	86 (83—89, *n* = 54)		0.717
Head circumference, cm, median (IQR)	48 (47—49, *n* = 49)	48 (46—49, *n* = 54)		0.557
Short-term outcome at term age or before discharge				
Survival without cerebral injury*, *n* (%)	58 (70.7)	60 (66.7)	1.06 (0.92—1.23)	0.432
Death, *n* (%)	10 (12.2)	10 (11.1)	1.10 (0.60—2.01)	0.762
No intraventricular hemorrhage, *n* (%)	65 (79.2)	68 (75.6)		0.488
Intraventricular hemorrhage I–II, *n* (%)	9 (11.0)	13 (14.4)		
Intraventricular hemorrhage III–IV, *n* (%)	8 (9.8)	9 (10.0)		
No cystic periventricular leukomalacia, *n* (%)	79 (96.3)	89 (98.9)		0.144
Cystic periventricular leukomalacia II, *n* (%)	2 (2.4)	–		
Cystic periventricular leukomalacia III, *n* (%)	1 (1.2)	1 (1.1)		
Culture proven early onset sepsis, *n* (%)	34 (41.5)	49 (54.4)	0.76 (0.55—1.05)	0.100
Necrotizing enterocolitis any grade**, *n* (%)	9 (11.0)	5 (5.6)	1.98 (0.62—6.28)	0.249
Bronchopulmonary dysplasia^†^, *n* (%)	32 (39.0)	35 (38.9)	1.00 (0.71—1.41)	0.984
Retinopathy of prematurity ≥ Grade 2, *n* (%)	17 (20.7)	28 (31.1)	0.67 (0.44—1.02)	0.062

* Defined as any intraventricular hemorrhage and/or cystic periventricular leukomalacia at term age or before discharge

† Defined as oxygen dependency or need of respiratory support at 36 weeks corrected age

** Medical and/or surgical intervention

Similarly, very preterm (28 + 0 to 31 + 6 weeks) neonates showed no differences in baseline demographic data at 2 years of corrected age, with a median (IQR) gestational age of 30.0 (28.9–31.0) weeks in the NIRS group and 29.9 (28.9–31.1) in the control group (*p* = 0.960; Supplementary Table 4). There was also no significant difference in survival without NND between the NIRS group and control group at 2 years of corrected age (Table 3). However, neonates in the NIRS group had significantly lower rates of cerebral palsy, visual impairment, and/or hearing impairment (Table 3) compared to those in the control group. In the NIRS group, there were significantly more neonates with retinopathy of prematurity (ROP) ≥ Grade 2 until term age or before discharge. However, at 2 years of corrected age, visual impairment was more frequent in the control group.

**Table 3:** Short-term and long-term outcome of preterm neonates with 28–31 weeks of gestation with and without monitoring of cerebral oxygen saturation to guide interventions during immediate transition after birth.

	NIRS-group *n* = 129	Control-group *n* = 116	Relative risk (95% CI)	*p*-value
Long-term outcome at 2 years corrected age				
Survival without moderate-to-severe NDD (*n* = 172), *n* (%)	90 (69.8)	82 (70.7)	0.98 (0.86—1.12)	0.748
Death or moderate-to-severe disability (*n* = 72), *n* (%)	39 (30.2)	33 (28.5)		
Postnatal accident with cerebral injury (*n* = 1), *n* (%)	–	1 (0.9)		–
Death (*n* = 3), *n* (%)	2 (1.6)	1 (0.9)		
Moderate-to-severe disability (*n* = 69), *n* (%)	37 (28.7)	32 (27.6)	1.04 (0.70—1.559	0.853
Cerebral palsy, yes/no, *n* (%)	1/112 (0.9)	6/103 (5.8)	0.15 (0.03—0.67)	0.013
Vision impairment, yes/no, *n* (%)	1/112 (0.9)	6/103 (5.8)	0.15 (0.05—0.49)	0.002
Corrected hearing loss/deaf, yes/no, *n* (%)	1/112 (0.9)	3/103 (2.9)	0.31 (0.12—0.77)	0.012
Cerebral palsy and/or vision impairment and/or corrected hearing loss/deaf, yes/no, *n* (%)	1/112 (0.9)	8/103 (7.8)	0.12 (0.03—0.45)	0.002
Bayleys cognitive score, median (IQR)	100 (95—110, *n* = 117)	100 (90—110, *n* = 104)		0.241
Bayleys language score, median (IQR)	97 (84—111, *n* = 78)	97 (91—111, *n* = 71)		0.548
Age corrected, months, median (IQR)	24 (24—25, *n* = 126)	24 (24—25, *n* = 115)		0.893
Weight, kg, median (IQR)	11.8 (10.6—12.8, *n* = 107)	11.7 (10.5—12.7, *n* = 98)		0.660
Height, cm, median (IQR)	87 (84—90, *n* = 104)	87 (84—90, *n* = 96)		0.840
Head circumference, cm, median (IQR)	50 (21—75, *n* = 98)	50 (16—70, *n* = 91)		0.568
Short term outcome at term age or before discharge				
Survival without cerebral injury*, *n* (%)	118 (91.5)	101 (87.1)	1.05 (0.97—1.14)	0.249
Death, *n* (%)	2 (1.6)	–		
No intraventricular hemorrhage, *n* (%)	119 (92.3)	101 (87.1)		0.163
Intraventricular hemorrhage I–II, *n* (%)	8 (6.2)	13 (11.2)		
Intraventricular hemorrhage III–IV, *n* (%)	2 (1.6)	2 (1.7)		
No cystic periventricular leukomalacia, *n* (%)	119 (92.3)	101 (87.1)		0.163
Cystic periventricular leukomalacia II, *n* (%)	8 (6.2)	13 (11.2)		
Cystic periventricular leukomalacia III, *n* (%)	2 (1.6)	2 (1.7)		
Culture proven early onset sepsis, *n* (%)	30 (23.3)	31 (26.7)	0.87 (0.67—1.12)	0.287
Necrotizing enterocolitis any grade**, *n* (%)	3 (2.3)	3 (2.6)	0.90 (0.74—1.09)	0.274
Bronchopulmonary dysplasia^†^, *n* (%)	5 (3.9)	4 (3.5)	1.12 (0.28—4.51)	0.869
Retinopathy of prematurity ≥ Grade 2, *n* (%)	6 (4.7)	2 (1.7)	2.70 (1.12—6.49)	0.027

* Defined as any intraventricular hemorrhage and/or cystic periventricular leukomalacia at term age or before discharge

† Defined as oxygen dependency or need of respiratory support at 36 weeks corrected age

** Medical and/or surgical intervention

## Discussion

The present 2-year follow-up study revealed that neonates born before 32 weeks of gestation, who underwent crSO_2_ monitoring and targeted interventions during immediate postnatal stabilization and resuscitation, showed no significant difference in overall survival without moderate-to-severe NDD compared to those receiving standard care. However, very preterm (28 + 0 to 31 + 6 weeks) neonates in the NIRS group had significantly lower rates of cerebral palsy, visual impairment, and hearing impairment compared to the control group receiving standard care alone.

Mortality in preterm neonates born before 32 weeks of gestation did not differ between the NIRS group and control group. Not surprisingly, mortality was higher in extremely preterm (<28 weeks) neonates than in very preterm (28 + 0 to 31 + 6 weeks) neonates. In this study, the overall mortality rate in very preterm neonates was comparable to previous findings, with a low mortality of approximately 1% (12–14). In extremely preterm (<28 weeks) neonates, the present mortality rate of approximately 12% was also rather low compared to the literature, which suggests an overall improvement in the outcomes of preterm neonates (12, 14, 15).

Similarly, the overall rates of moderate-to-severe NDD were not significantly different between the NIRS group and control group, and, as expected, the rate of moderate-to-severe NDD was higher in extremely preterm (<28 weeks) neonates than in very preterm (28 + 0 to 31 + 6 weeks) neonates. In very preterm neonates, the rates of moderate-to-severe NDD were slightly higher than those in previously published data (16, 17). In extremely preterm neonates, the observed rates of moderate-to-severe NDD were comparable to those reported in Europe and North America, with similar survival rates (15).

In the present study, the BSID is mainly used for the 2-year neurodevelopmental assessment (18, 19). The limitations of this assessment modality are the dependency on the compliance of the child, which itself depends on the assessor. Alternatives include parental questionnaires, the limitations of which are the subjective assessments by parents. However, a comparable parental questionnaire to the BSID tests is the PARCA-R, which evaluates cognitive and language development at 2 years of age (20). In the present study, only 23 neonates were tested using PARCA-R, but these neonates had this in addition to a BSID test; therefore, the results of PARCA-R were not included in the analyses. The BSID scores in the present study were overall high for extremely (<28 weeks) and very (28 + 0 to 31 + 6 weeks) preterm neonates. Similarly, the short-term outcome of survival without cerebral injury was higher than expected in the COSGOD III trial (11). We speculate that the favorable short- and long-term outcomes may be attributed to an overall improvement in outcomes for preterm neonates. However, this may be accounted for to some degree by selection bias, as most centers that included neonates required antenatal consent; consequently, neonates with higher risk due to imminent birth were often not enrolled (11).

No overall difference in cerebral palsy rates was observed between the NIRS group and control group, with rates comparable to those reported in the literature, particularly among extremely preterm (<28 weeks) neonates (21, 22). In very preterm (28 + 0 to 31 + 6 weeks) neonates, we observed significantly lower cerebral palsy rates in the NIRS group than in the control group. However, owing to the low absolute number of cases, this significant finding should be interpreted with caution.

Concerning visual impairment, a significant difference was noted between the two groups in the cohort of very preterm neonates, with the NIRS group experiencing less visual impairment. Paradoxically, the rate of ROP was significantly higher in the NIRS group, which suggests a higher number of visual impairments in this group. This seemingly contradictory finding might be explained by the fact that screening strategies involving the early identification and appropriate treatment of children with ROP prevent blindness (23, 24). Furthermore, in countries where survival following premature or complicated birth has improved in the last decade, an increase in visual morbidity has been observed due to cerebral visual impairment (25). In addition, the results must again be interpreted with caution due to the overall low numbers, which are in accordance with data published from Western Europe and North America with a reduction in the proportion of moderately preterm children developing ROP (26, 27).

Only a few neonates in the present cohort developed hearing impairment, with significant differences between the two groups, with more frequent occurrences in the control group. The incidence was comparable to that reported in the literature (21). Although this finding is statistically significant, it should be interpreted with caution due to the limited sample size of neonates; therefore, further studies are necessary to investigate this association more comprehensively.

However, it is remarkable that if cerebral palsy, visual impairment, and hearing impairment are considered together, there is a reasonable difference between the NIRS group and control group in very preterm (28 + 0 to 31 + 6 weeks) neonates, suggesting clinical relevance. We can only speculate on the pathophysiological pathway for a possible improvement of cerebral palsy, visual impairment, and hearing impairment in very preterm (28 + 0 to 31 + 6 weeks) neonates. First, the improvement might be due to improved tissue oxygenation in these very preterm (28 + 0 to 31 + 6 weeks) neonates during the immediate transition. Second, interventions based on crSO_2_ monitoring may have improved end-organ perfusion (28).

In the NIRS group, fewer neonates had culture-proven sepsis than in the control group. Because neonatal sepsis is known to be associated with a higher risk of long-term morbidities, particularly cerebral palsy (29, 30), we adjusted for culture-proven sepsis. However, this adjustment did not alter the statistical significance of the differences between the groups.

### Strengths and limitations

The strength of this present 2-years follow-up study lies in its multinational, multicenter design, featuring a large cohort of preterm neonates born before 32 weeks of gestation and achieving a follow-up rate of nearly 80%. In addition, most neonates underwent standardized 2-year follow-up assessments in an outpatient clinic with formal testing (BSID test). However, the study also has limitations, as its retrospective design, owing to limited financial support for a prospective follow-up study, introduces potential bias. This might have resulted in a higher lost-to-follow-up rate and more informal assessments, and some neonates, although few, had only informal outcome assessments. Furthermore, the lost-to-follow-up group included a higher proportion of neonates with greater gestational age than the analyzed cohort, which may have introduced a selection bias. However, neonates with a lower gestational age, who are of relevance for long-term outcomes, were more strongly represented in the final analyses. Due to the retrospective study design and loss to follow-up, differences in maternal and fetal characteristics and interventions were also observed. As the impact of the most significant maternal and fetal differences on long-term outcomes is uncertain and adjustment was not feasible for some variables, such as “other” causes of preterm birth, only significant neonatal differences were addressed in the gestational age-based subgroup analyses and by adjustment for short-term neonatal infectious morbidities.

## Conclusion

The present 2-year follow-up study on preterm neonates born before 32 weeks of gestation revealed that monitoring of crSO_2_, along with dedicated interventions during immediate postnatal transition, did not improve overall survival without moderate-to-severe NND compared to standard care. However, very preterm neonates (28 + 0 to 31 + 6 weeks) in the NIRS group had significantly lower rates of cerebral palsy, visual impairment, and hearing impairment than those in the control group, who received standard care alone. These findings should be viewed as hypothesis-generating, indicating that crSO₂ monitoring, along with dedicated interventions during stabilization and resuscitation immediately after birth, could provide clinical benefits for this group, particularly concerning long-term neurodevelopmental outcomes.

## Data Availability

The raw data supporting the conclusions of this article will be made available on demand by the authors, without undue reservation.

## COSGOD III study group

The following investigators, in addition to those listed as authors, participated in this trial as collaborators:

Division of Neonatology, Department of Pediatrics, Medical University of Graz, Graz, Austria: Elisabeth Pichler-Stachl, PhD, Marlies, Bruckner, MD, Corinna, Binder-Heschl, PhD, Daniel Pfurtscheller, MD, Johann Martensen, MD, Nina Höller, MD, Evelyn Ziehenberger.

Comprehensive Center for Pediatrics, Department of Pediatrics and Adolescent Medicine, Division of Neonatology, Intensive Care and Neuropediatrics, Medical University of Vienna, Vienna, Austria: Angelika Berger, MBA (local PI), Sigrid Baumgartner, MD, Agnes Grill, MD, Michaela Mayr, MD, Judith Rittenschober-Boehm, PhD, Michael Schneider, MD, Julia Buchmayer, MD.

Department of Pediatrics II, Neonatology, Medical University of Innsbruck, Innsbruck, Austria: Christina Schreiner, PhD, Elke Griesmaier, PhD, Vera Neubauer, PhD, Peter Wöckinger, MD, Anna Posod, PhD.

NICU, Department for Perinatology, Division of Gynaecology and Obstetrics, University Medical Centre Ljubljana, Slovenia: Anja Marolt, RN, Ana Dimnik, RN, Vlasta Lubej Kurtovič, RN.

Infant Centre, University College Cork, Cork University Maternity Hospital, Cork, Ireland: Christoph E. Schwarz, MD, Aisling Garvey, MD, Jurate Panaviene, MD, David Healy, MD, Nahla Ahmed, MD. Ita Herlighy, RN.

Department of Neonatology, University Children's Hospital of Tübingen, Germany: Axel R. Franz, MD, Kerstin Gründler, MD.

Neonatologia e Terapia Intensiva Neonatale (TIN) Ospedale dei Bambini “V.Buzzi,” Milano, Italia: Francesca Castoldi, MD, Francesco Cavigioli, MD.

II Department of Neonatology, Neonatal Biophysical Monitoring and Cardiopulmonary Therapies Research Unit, Chair of Neonatology, Poznan University of Medical Sciences, Poznan, Poland: Lukasz Karpinski, PhD, Zuzanna Kozłowska, MD, Marcin Minta, MD, Zuzanna Owsiańska, PhD, Sonia Kahtan, PhD, Natalia Neumann-Klimasińska, PhD, Karolina Wróbel, MD, Agata Kubiaczyk, MD, Katarzyna Kosik, MD, Katarzyna Olek, MD, Michalina Bugiera, MD PhD, Julita Porwolik, MD, Agnieszka Basiukajć, MD, Elżbieta Czapla, MSc, Wojciech Łukaszuk, BS, Katarzyna Gryczka, MD, Dobrochna Naskręcka, MD, Jan Mazela, PhD, Marta Szymankiewicz-Bręborowicz, PhD.

Division of Neonatology and Pediatric Intensive Care Medicine, Center for Pediatrics and Adolescent Medicine, Medical Center - University of Freiburg, Faculty of Medicine, University of Freiburg, Freiburg, Germany: Daniel Klotz, MD, Jana Baumgartner, MD.

Institute for Maternal and Child Health, “IRCCS Burlo Garofolo”, Trieste, Italy: Stefano Bembich, PhD, Laura Travan, MD.

Department of Pediatrics, University of Alberta, Edmonton, Alberta, Canada: Brenda Law, MSc, Po-Yin Cheung, PhD, Georg M Schmölzer, PhD.
