# Supplementary material for: Two-year neurodevelopmental outcome in preterm neonates with cerebral oxygenation monitoring after birth: a multinational, multicenter retrospective follow-up study of the COSGOD III trial
Source: Front Pediatr. 2026 Jun 15;14:1754084. doi: 10.3389/fped.2026.1754084 (PMC13312903; doi:10.3389/fped.2026.1754084)
Supplement: Supplementary file 1 [file Table1.docx]

**Supplemental Table 1:** Maternal, fetal, and neonatal baseline characteristics of preterm neonates, who were included and who were lost to follow up.

Lost to

Follow up follow up

n = 417 n = 109 p-value

*Maternal cause of preterm birth*

Antepartum bleeding (n=415/106), n (%) 53 (12.8) 9 (8.5) .449

Chorioamnionitis (n=415/106), n (%) 103 (24.8) 22 (20.7) .368

Premature rupture of membranes (n=416/106), n (%) 124 (29.8) 38 (35.9) .191

Preeclampsia (n=415/107), n (%) 77 (18.6) 20 (18.7) .968

Gestational diabetes (n=415/106), n (%) 10 (2.4) 7 (6.6) .011

Others (n=414/105), n (%) 93 (22.5) 14 (13.3) .013

*Fetal cause of preterm birth*

Intrauterine growth restriction (n=413/107), n (%) 78 (18.9) 19 (17.8) .775

Fetal bradycardia (n=413/108), n (%) 70 (17.0) 19 (17.6) .843

Pathological doppler sonography (n=413/107), n (%) 76 (18.4) 20 (18.7) .945

Multiples (n=412/106), n (%) 50 (12.1) 9 (8.5) .384

Others (n=413/105), n (%) 19 (4.6) 7 (6.7) .308

Lost to

Follow up follow up

n = 417 n = 109 p-value

*Mode of delivery (n=416/108)*

Spontaneous vaginal delivery, n (%) 54 (13.0) 15 (13.9) .951

Caesarean section, n (%) 361 (86.8) 92 (85.2)

Instrumental delivery, n (%) 1 (0.2) 1 (0.9)

*Cord clamping time (n=387/104)*

<30 seconds, n (%) 254 (65.6) 55 (52.9) .143

30-60 seconds, n (%) 81 (20.9) 32 (30.8)

>60 seconds, n (%) 52 (13.4) 17 (16.4)

*Neonatal characteristics*

Gestational age, weeks, median (IQR) 28.6 (26.4-30.4) 30.1 (28.0-31.1) <.001

Gestational age <28weeks, n (%) 172 (41.3) 26 (23.9) .004

Gestational age >28 weeks, n (%) 245 (58.8) 83 (76.2)

Birth weight, gram, median (IQR) 1060 (800-1316) 1210 (945-1530) <.001

Male/female (n=302/301), n (%) 223/190(54.0/46.0) 54/55 (49.5/50.5) .338

Umbilical artery pH, median (IQR) 7.33 (7.28-7.37) 7.32 (7.29-7.36) .173

Apgar 1, median (IQR) 7.0 (5.0-8.0) 7.0 (6.0-8.0) .044

Apgar 5, median (IQR) 8.0 (8.0-9.0) 8.0 (7.5-9.0) .991

Apgar 10, median (IQR) 9.0 (8.0-9.0) 9.0 (9.0-9.0) .743
