# Supplementary material for: Two-year neurodevelopmental outcome in preterm neonates with cerebral oxygenation monitoring after birth: a multinational, multicenter retrospective follow-up study of the COSGOD III trial
Source: Front Pediatr. 2026 Jun 15;14:1754084. doi: 10.3389/fped.2026.1754084 (PMC13312903; doi:10.3389/fped.2026.1754084)
Supplement: Supplementary file 2 [file Table2.docx]

**Supplemental Table 2a: Maternal, fetal, and neonatal baseline characteristics of preterm neonates < 32 weeks of gestation with and without monitoring of cerebral oxygen saturation to guide interventions during immediate transition after birth**

NIRS-group Control-group

n = 211 n = 206 p-value

*Maternal cause of preterm birth*

Antepartum bleeding, n (%) 28 (13.3) 25 (12.2) .810

Chorioamnionitis, n (%) 42 (20.0) 61 (29.8) .007

Premature rupture of membranes, n (%) 65 (31.0) 59 (28.6) .600

Preeclampsia, n (%) 38 (18.1) 39 (19.0) .766

Gestational diabetes, n (%) 3 (1.4) 7 (3.4) .158

Others, n (%) 40 (19.1) 53 (26.0) .041

*Fetal cause of preterm birth*

Intrauterine growth restriction, n (%) 37 (17.7) 41 (20.1) .342

Fetal bradycardia, n (%) 42 (20.1) 28 (13.7) .022

Pathological doppler sonography, n (%) 43 (20.6) 33 (16.2) .184

Multiples, n (%) 29 (13.9) 21 (10.3) .272

Others, n (%) 6 (2.9) 13 (6.4) .142

*Mode of delivery*

Spontaneous vaginal delivery, n (%) 33 (15.7) 21 (10.2) .072

Caesarean section, n (%) 176 (83.8) 185 (89.8)

Instrumental delivery, n (%) 1 (0.5) -

*Cord clamping time*

< 30 seconds, n (%) 129 (64.8) 125 (66.5) .037

30 – 60 seconds, n (%) 47 (23.6) 34 (18.1)

> 60 seconds, n (%) 23 (11.6) 29 (15.4)

*Neonatal characteristics*

Gestational age, weeks, median (IQR) 28.7 (26.9 – 30.6) 28.4 (26.3 – 30.1) .138

Gestational age < 28 weeks, n (%) 82 (38.9) 90 (43.7) .378

Gestational age > 28 weeks, n (%) 129 (61.1) 116 (56.3)

Birth weight, gram, median (IQR) 1100 (830 – 1360) 1000 (780 – 1285) .053

Male/female, n (%) 109/100 (52.2/47.9) 114/90 (56.2/44.1) .306

Umbilical artery pH, median (IQR) 7.33 (7.28 – 7.37) 7.33 (7.28 – 7.37) .119

Apgar 1, median (IQR) 7.0 (5.0 – 8.0) 7.0 (5.0 – 8.0) .752

Apgar 5, median (IQR) 8.0 (8.0 – 9.0) 8.0 (8.0 – 9.0) .679

Apgar 10, median (IQR) 9.0 (8.0 – 9.0) 9.0 (8.0 – 9.0) .125

**Supplemental Table 2b: Interventions during first 15 minutes after birth and the first 24 hours after birth of preterm neonates < 32 weeks of gestation with and without monitoring of cerebral oxygen saturation to guide interventions during immediate transition after birth**

NIRS-group Control-group p-value

n = 211 n = 206

*First 15 minutes after birth*

Supplemental oxygen, n (%) 208 (98.6) 197 (95.1) .085

No respiratory support, n (%) 2 (1.0) 3 (1.5) .680

Mask continuous positive pressure, n (%) 76 (36.0) 74 (35.9)

Mask positive pressure ventilation, n (%) 108 (51.2) 111 (53.9)

Intubation, n (%) 25 (11.9) 18 (8.7)

Chest compressions, n (%) 3 (1.4) 3 (1.5) .975

Caffeine, n (%) 58 (27.9) 74 (36.1) .061

Adrenaline, n (%) 2 (1.0) - -

Surfactant, n (%) 20 (9.5) 22 (10.7) .519

Intravenous volume, n (%) 10 (4.7) 1 (0.5) .025

Others, n (%) 5 (2.4) 3 (1.5) .383

*First 24 hours after birth*

Surfactant, n (%) 117 (55.7) 117 (56.8) .759

No respiratory support, n (%) 13 (6.2) 11 (5.3) .220

Non-invasive ventilation, n (%) 142 (67.3) 153 (74.3)

Mechanical ventilation, n (%) 56 (26.5) 42 (20.4)
