# Supplementary material for: Two-year neurodevelopmental outcome in preterm neonates with cerebral oxygenation monitoring after birth: a multinational, multicenter retrospective follow-up study of the COSGOD III trial
Source: Front Pediatr. 2026 Jun 15;14:1754084. doi: 10.3389/fped.2026.1754084 (PMC13312903; doi:10.3389/fped.2026.1754084)
Supplement: Supplementary file 3 [file Table3.docx]

**Supplemental Table 3a: Maternal, fetal, and neonatal baseline characteristics of preterm neonates < 28 weeks of gestation with and without monitoring of cerebral oxygen saturation to guide interventions during immediate transition after birth**

NIRS-group Control-group p-value

n = 82 n = 90

*Maternal cause of preterm birth*

Antepartum bleeding, n (%) 13 (16.1) 20 (22.5) .297

Chorioamnionitis, n (%) 25 (30.9) 35 (39.9) .255

Premature rupture of membranes, n (%) 33 (40.7) 31 (34.4) .475

Preeclampsia, n (%) 12 (14.8) 13 (14.6) .958

Gestational diabetes, n (%) 2 (2.5) - -

Others, n (%) 13 (16.1) 26 (29.2) .070

*Fetal cause of preterm birth*

Intrauterine growth restriction, n (%) 9 (11.1) 13 (14.8) .486

Fetal bradycardia, n (%) 17 (20.1) 14 (15.9) .383

Pathological doppler sonography, n (%) 15 (18.5) 12 (13.6) .406

Multiples, n (%) 5 (6.2) 9 (10.2) .452

Others, n (%) 1 (1.2) 7 (8.0) .046

*Mode of delivery*

Spontaneous vaginal delivery, n (%) 14 (17.1) 10 (11.1) .238

Caesarean section, n (%) 68 (82.9) 80 (88.9)

Instrumental delivery, n (%) - -

*Cord clamping time*

< 30 seconds, n (%) 46 (59.0) 57 (67.9) .716

30 – 60 seconds, n (%) 18 (23.1) 17 (20.2)

> 60 seconds, n (%) 14 (18.0) 10 (11.9)

*Neonatal characteristics*

Gestational age, weeks, median (IQR) 26.3 (25.3 – 27.1) 26.1 (25.3 – 26.9) .345

Birth weight, gram, median (IQR) 810 (640 – 950) 782 (650 – 890) .254

Male/female, n (%) 44/37(54.3/45.7) 56/32 (63.6/36.4) .370

Umbilical artery pH, median (IQR) 7.34 (7.29 – 7.38) 7.34 (7.28 – 7.39) .705

Apgar 1, median (IQR) 7.0 (5.0 – 8.0) 6.0 (5.0 – 8.0) .313

Apgar 5, median (IQR) 8.0 (7.0 – 9.0) 8.0 (7.0 – 9.0) .373

Apgar 10, median (IQR) 9.0 (8.0 – 9.0) 9.0 (8.0 – 9.0) .513

**Supplemental Table 3b: Interventions during first 15 minutes after birth and the first 24 hours after birth** **of preterm neonates < 28 weeks of gestation with and without monitoring of cerebral oxygen saturation to guide interventions during immediate transition after birth**

NIRS-group Control-group p-value

n = 82 n = 90

*First 15 minutes after birth*

Supplemental oxygen, n (%) 81 (98.8) 86 (95.6) .107

No respiratory support, n (%) 1 (1.2) 1 (1.1) .587

Mask continuous positive pressure, n (%) 22 (26.8) 28 (31.1)

Mask positive pressure ventilation, n (%) 44 (53.7) 45 (50.0)

Intubation, n (%) 15 (18.3) 16 (17.8)

Chest compressions, n (%) - 1 (1.1) -

Caffeine, n (%) 19 (23.5) 29 (32.2) .289

Adrenaline, n (%) - - -

Surfactant, n (%) 14 (17.1) 16 (17.8) .895

Intravenous volume, n (%) 4 (4.9) 1 (1.5) .219

Others, n (%) 4 (4.9) - -

*First 24 hours after birth*

Surfactant, n (%) 51 (62.2) 59 (65.6) .594

No respiratory support, n (%) 6 (7.3) 1 (1.1) .334

Non-invasive ventilation, n (%) 46 (56.1) 54 (60.0)

Mechanical ventilation, n (%) 30 (36.6) 35 (38.9)
