# Supplementary material for: Two-year neurodevelopmental outcome in preterm neonates with cerebral oxygenation monitoring after birth: a multinational, multicenter retrospective follow-up study of the COSGOD III trial
Source: Front Pediatr. 2026 Jun 15;14:1754084. doi: 10.3389/fped.2026.1754084 (PMC13312903; doi:10.3389/fped.2026.1754084)
Supplement: Supplementary file 4 [file Table4.docx]

**Supplemental Table 4a: Maternal, fetal, and neonatal baseline characteristics of preterm neonates with 28 to 31 weeks of gestation with and without monitoring of cerebral oxygen saturation to guide interventions during immediate transition after birth**

NIRS-group Control-group p-value

n = 129 n = 116

*Maternal cause of preterm birth*

Antepartum bleeding, n (%) 15 (11.6) 5 (4.3) .093

Chorioamnionitis, n (%) 17 (13.2) 26 (22.8) .026

Premature rupture of membranes, n (%) 32 (24.8) 28 (24.1) .910

Preeclampsia, n (%) 26 (20.2) 26 (22.4) .601

Gestational diabetes, n (%) 1 (0.8) 7 (6.0) .061

Others, n (%) 27 (20.9) 27 (23.5) .414

*Fetal cause of preterm birth*

Intrauterine growth restriction, n (%) 28 (21.2) 28 (24.1) .514

Fetal bradycardia, n (%) 25 (19.5) 14 (12.1) .109

Pathological doppler sonography, n (%) 28 (21.9) 21 (18.1) .366

Multiples, n (%) 24 (18.8) 12 (10.4) <.001

Others, n (%) 5 (3.9) 6 (5.2) .640

*Mode of delivery*

Spontaneous vaginal delivery, n (%) 19 (14.8) 11 (9.5) .124

Caesarean section, n (%) 108 (84.4) 105 (90.5)

Instrumental delivery, n (%) 1 (0.8) -

*Cord clamping time*

< 30 seconds, n (%) 83 (68.6) 68 (65.4) .059

30 – 60 seconds, n (%) 29 (24.6) 17 (16.4)

> 60 seconds, n (%) 9 (7.4) 19 (18.3)

*Neonatal characteristics*

Gestational age, weeks, median (IQR) 30.0 (28.9 – 31.0) 29.9 (28.9 – 31.1) .960

Birth weight, gram, median (IQR) 1300 (1100 – 1495) 1240 (1083 – 1500) .372

Male/female, n (%) 65/63(50.8/49.2) 58/58 (50.0/50.0) .875

Umbilical artery pH, median (IQR) 7.31 (7.27 – 7.36) 7.33 (7.29 – 7.36) .028

Apgar 1, median (IQR) 7.0 (6.0 – 8.0) 7.0 (6.0 – 8.0) .384

Apgar 5, median (IQR) 8.0 (8.0 – 9.0) 9.0 (8.0 – 9.0) .161

Apgar 10, median (IQR) 9.0 (8.0 – 9.0) 9.0 (9.0 – 9.0) .322

**Supplemental Table 4b: Interventions during first 15 minutes after birth and the first 24 hours after birth of preterm neonates with 28 to 31 weeks of gestation with and without monitoring of cerebral oxygen saturation to guide interventions during immediate transition after birth**

NIRS-group Control-group p-value

n = 129 n = 116

*First 15 minutes after birth*

Supplemental oxygen, n (%) 127 (98.5) 112 (96.6) .224

No respiratory support, n (%) 1 (0.8) 2 (1.7) .655

Mask continuous positive pressure, n (%) 54 (41.9) 46 (39.7)

Mask positive pressure ventilation, n (%) 64 (49.6) 66 (56.9)

Intubation, n (%) 10 (7.8) 2 (1.7)

Chest compressions, n (%) 3 (2.3) 2 (1.8) .762

Caffeine, n (%) 39 (30.7) 45 (39.1) .029

Adrenaline, n (%) 2 (1.6) 0 (-) -

Surfactant, n (%) 6 (4.7) 6 (5.2) .800

Intravenous volume, n (%) 6 (4.7) - -

Others, n (%) 1 (0.8) 3 (2.6) .027

*First 24 hours after birth*

Surfactant, n (%) 66 (51.6) 58 (50.0) .656

No respiratory support, n (%) 7 (5.4) 10 (8.6) .001

Non-invasive ventilation, n (%) 96 (74.4) 99 (85.3)

Mechanical ventilation, n (%) 26 (20.2) 7 (6.0)
